# Interpenetration of polymeric microgels at ultrahigh densities

**DOI:** 10.1038/s41598-017-01471-3

**Published:** 2017-05-03

**Authors:** Priti S. Mohanty, Sofi Nöjd, Kitty van Gruijthuijsen, Jérôme J. Crassous, Marc Obiols-Rabasa, Ralf Schweins, Anna Stradner, Peter Schurtenberger

**Affiliations:** 1 Physical Chemistry, Department of Chemistry, Lund University, 221 00 Lund, Sweden; 2 School of Applied Sciences, KIIT University, Bhubaneswar, 751024 India; 3 Adolphe Merkle Institute, University of Fribourg, Fribourg, 1700 Switzerland; 4 Large Scale Structures Group, Institut Laue-Langevin, Grenoble, 38042 France

## Abstract

Soft particles such as polymeric microgels can form ultra-dense phases, where the average center-to-center distance *a*_s_ can be smaller than the initial unperturbed particle diameter *σ*_0_, due to their ability to interpenetrate and compress. However, despite of the effort devoted to microgels at ultrahigh densities, we know surprisingly little about their response to their environment at effective volume fractions *ϕ*_*eff*_ above close packing (*ϕ*_*cp*_), and the existing information is often contradictory. Here we report direct measurements of the size and shape of poly(N-isopropylacrylamide) microgels at concentrations below and above *ϕ*_*cp*_ using the zero average contrast method in small-angle neutron scattering. We complement these experiments with measurements of the average interparticle distances using small-angle x-ray scattering, and a determination of the glass transition using dynamic light scattering. This allows us to unambiguously decouple interaction effects from density-dependent variations of the particle size and shape at all values of *ϕ*_*eff*_. We demonstrate that the microgels used in this study significantly interpenetrate and thus change their size and shape only marginally even for *ϕ*_*eff*_ ≫ *ϕ*_*cp*_, a finding that may require changes in the interpretation of a number of previously published studies on the structural and dynamic properties of dense soft particle systems.

## Introduction

Microgel particles are intriguing soft and responsive colloids formed by a cross-linked polymeric structure that can undergo reversible continuous or discontinuous volume transitions upon variations of external stimuli such as temperature, pH or ionic strength^1–3^. As a result of their soft interaction potential, they can form dispersions with ultra-high densities at effective packing fractions *ϕ*_*eff*_ far above close packing (*ϕ*_*cp*_), where the average center-to-center distance *a*_*s*_ is smaller than the initial unperturbed particle diameter *σ*_0_, and which possess a number of intriguing properties. Here *ϕ*_*eff*_ is defined as *ϕ*_*eff*_ = *n*_*p*_ *πσ* ^3^/6, where *n*_*p*_ is the number density of the particles, and *σ* their diameter. In particular thermoresponsive particles such as poly(N-isopropylacrylamide) (PNIPAm)-based microgels have been used recently as seemingly ideal model systems to investigate various “hot” topics in the physics of crystallisation and melting, dynamical arrest, glass transition and jamming^4–8^. These microgel-based investigations have often relied on the ability of the particles to vary their size as a function of temperature, and used temperature as a convenient way to tune the particle size and thus the volume fraction. This has led to a number of exciting findings such as the existence of a thermal vestige of the zero-temperature jamming transition^7^, or the applicability of the concept of fragility used in molecular glass formers to soft colloids^9^.

Given the fact that microgels can in principle adapt both their size as well as their shape in response to the environment, any attempt to understand the structural and dynamic properties of dense microgel dispersions thus requires quantitative information about the behaviour of the individual particle as a function of temperature and density. This can for example be illustrated with recent work on soft glasses and pastes, which are fascinating materials that offer quite unique flow properties. Attempts to create widely applicable models for soft glasses and pastes were based on the assumption that particles compress and facet beyond close packing^10^. A compression of the microgel size above close packing was also postulated based on static light scattering measurements of soft particle suspensions and glasses^9^. Systematic small-angle neutron scattering (SANS) experiments^11^ on the other hand seemed to indicate that PNIPAm microgels decrease in size with increasing concentration already before reaching close packing, while their interaction potential appeared not to change with temperature below the collapse temperature of 32 °C, and could be well described by an effective hard sphere potential.

Despite the large number of publications dealing with dense soft particle suspensions, glasses and gels, there is indeed a surprising lack of information about the evolution of the size and shape of soft microgels with increasing concentration at and above close packing. This obviously has to do with the experimental difficulties connected to performing such measurements in a completely non-invasive and *in-situ* manner for *ϕ*_*eff*_ ≥ *ϕ*_*cp*_. While confocal laser scanning microscopy (CLSM) is routinely used to determine key properties such as pair correlation functions *g*(*r*) or particle mean square displacements 〈Δ*r* ^2^(*t*)〉 in this concentration regime^6^, it lacks sufficient spatial resolution to reveal subtle changes in the particle size and shape. SANS and SAXS are indeed capable of measuring structural changes with the necessary almost atomistic resolution, and previous SANS studies with PNIPAm microgels at low volume fractions have provided in-depth information about their structural properties. They have for example revealed that the polymer density inside the microgel particle in the swollen state is not homogeneous, but gradually decays from the centre towards the surface^12^. However, at higher effective volume fractions, the normalised scattering intensity or differential scattering cross section *dσ*(*q*)/*d*Ω obtained by these techniques generally contains contributions from both single particle structure (i.e. the particle form factor *P*(*q*)) and interparticle correlations (the structure factor *S*(*q*)) and is given by *dσ*(*q*)/*d*Ω ∝ *n*_*p*_Δ*ρ* ^2^ *P*(*q*)*S*(*q*), where *q* *=* (4*π*/*λ*)sin(*θ*/2) is the magnitude of the scattering vector and Δ*ρ* is the overall excess scattering length density of the particles. Changes in the conformation of the microgels could thus only be determined indirectly from the density-dependent investigations of the structure factor^11^. It is for this reason that we now report on an investigation of their structure at high effective volume fractions using small-angle neutron scattering (SANS) experiments with mixtures of deuterated and hydrogenated microgels under so-called zero average contrast (ZAC) conditions to extract the particle form factor at all densities^13, 14^.

The ZAC method relies on a 50–50 mixture (by number) of otherwise identical hydrogenated and deuterated particles at zero contrast conditions, i.e., in a mixture of H_2_O and D_2_O that has exactly the average scattering contrast of the particles. In a mixture of particles under ZAC condition, *dσ*(*q*)/*d*Ω is given by the sum of the partial scattering functions, which are divided into their self (I) and distinct (II) contributions^14, 15^

1 $$\frac{d\sigma }{d{\rm{\Omega }}}(q)\propto {\rm{\Delta }}{\rho }_{z}^{2}({S}_{D}^{I}+{S}_{H}^{I}+{S}_{DD}^{II}+{S}_{HH}^{II}-2{S}_{DH}^{II})$$

where Δ*ρ*_*z*_ = Δ*ρ*_*D*_ = −Δ*ρ*_*H*_. Note that in this notation ${S}_{D}^{I}$ and ${S}_{H}^{I}$ are directly related to the form factors of the deuterated and hydrogenated particles, respectively, with *S* ^*I*^ ∝ *n*_*p*_ *P*(*q*). If we assume that the scattering function of H and D particles and the interactions between H and D particles are identical, i.e., ${S}_{D}^{I}$ = ${S}_{H}^{I}$ and ${S}_{DD}^{II}$ = ${S}_{HH}^{II}$ = ${S}_{DH}^{II}$, *dσ*(*q*)/*d*Ω then reduces to

2 $$\frac{d\sigma }{d{\rm{\Omega }}}(q)\propto {\rm{\Delta }}{\rho }_{z}^{2}2{S}_{D}^{I}\propto {n}_{p}{\rm{\Delta }}{\rho }_{z}^{2}P(q)$$

In the remainder of the manuscript we demonstrate that the ZAC method indeed allows for a direct measurement of *P*(*q*) for microgels at concentrations below and above close packing, since contributions from interparticle interactions cancel. These experiments are then complemented with measurements of *S*(*q*) using small-angle x-ray scattering (SAXS), where both particles to a good approximation have the same scattering contrast. This provides us with a means to unambiguously decouple interaction effects from density-dependent variations of the particle size and shape at all effective volume fractions, and at two temperatures where the particles exhibit a different degree of swelling. We show that the particles hardly change their size even at concentrations *ϕ*_*eff*_ ≫ *ϕ*_*cp*_, and instead significantly interpenetrate. We finally discuss these results in view of previously published results, and point out the consequences of the strong particle interpenetration for the resulting structural and dynamic properties at these ultra-high packing fractions.

## Results

### Initial particle properties

**Figure 1:**
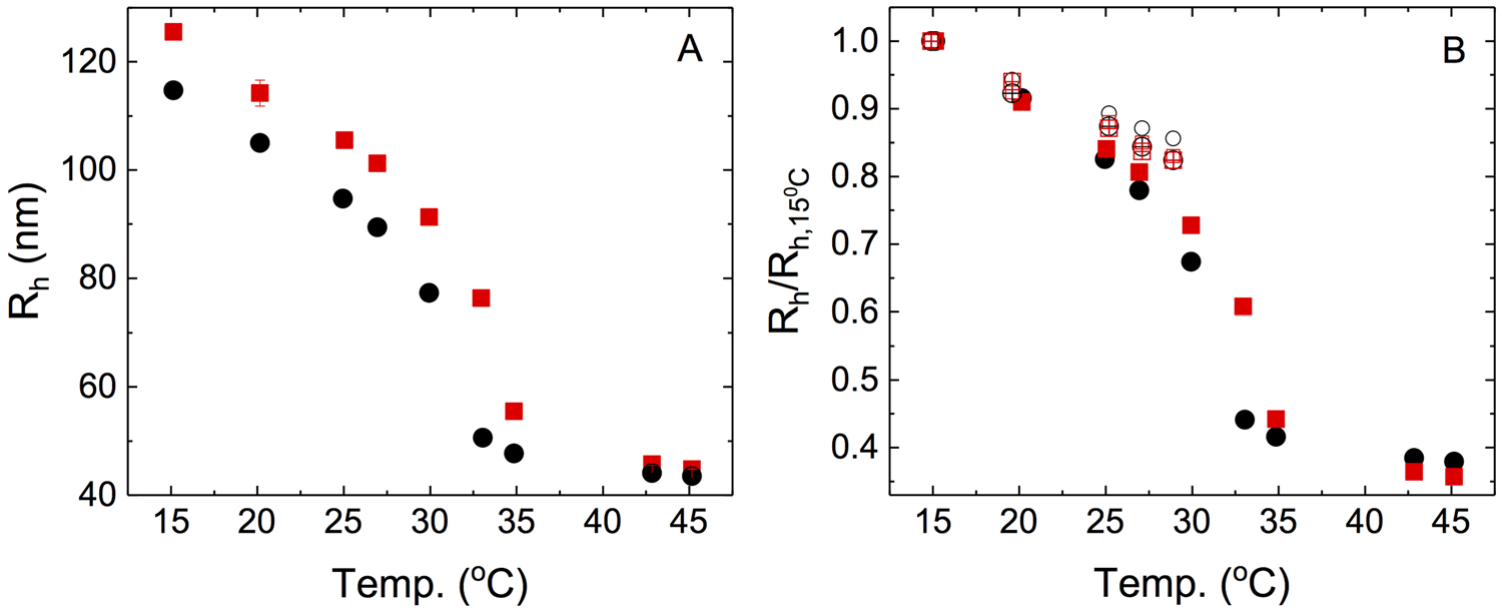
Swelling behavior of microgels. Temperature dependence of the hydrodynamic radius *R*_*h*_ for the deuterated and hydrogenated particles in H_2_O. Shown are the actual values (**A**) as well as rescaled values (**B**) where the temperature-dependent values *R*_*h*_(*T*) are normalized by *R*_*h*_(15 °C) as a function of temperature for deuterated (squares) and hydrogenated (circles) particles. Also shown are the corresponding values obtained in the ZAC solvent for Hm (open black circles) and Dm (open red squares), as well as the results obtained in 12:88 H_2_O:D_2_O, (Hm: open black crossed circles, Dm: open red crossed squares). Error bars are calculated based on the standard deviation of the measurements of the decay time for the individual scattering angles, and the resulting statistical error in the calculation of the average hydrodynamic radius from the *q* ^2^-dependence of these average decay times as described in the methods section. Note that for most data points the resulting error bars are smaller than the symbol size.

Hydrogenated (Hm) and deuterated (Dm) PNIPAm particles were synthesised such as to exhibit identical sizes and swelling properties using precipitation polymerization as described in the methods section. For this reason we choose molar fractions of cross-linker of 3.5 mol% for the hydrogenated and 5.4 mol% for the deuterated particles. The size and swelling behaviour of the particles were then assessed by dynamic light scattering (DLS), where Dm exhibits a weak isotope effect for the collapse transition temperature or volume phase transition temperature *T*_*VPT*_ in H_2_O. (see Fig. 1). However, we find a virtually identical temperature-dependent swelling behavior for a range of temperatures 15 ≤ *T* ≤ 27 °C, which was subsequently used for the evaluation of the effect of particle density on size and shape. The particles have hydrodynamic radii *R*_*h*_ of about 115 nm for the hydrogenated and 125 nm for the deuterated particles in the swollen form at *T* = 15 °C, and about 44 nm for the hydrogenated and 45 nm for the deuterated particles in the collapsed state at *T* > *T*_*VPT*_, i.e. a degree of swelling defined as (*R*_*h*_[15 °C]/*R*_*h*_[45 °C])^3^ ≈ 20 that is typical for this cross-linking density. Their properties are summarised in the methods section in Table 1. Addition of D_2_O makes the particle suspensions more prone to aggregation at high temperatures close to and above *T*_*VPT*_. However, since isotope effects mainly influence *T*_*VPT*_ and the swelling behaviour of the particles close to *T*_*VPT*_, we used the swelling curve in pure H_2_O in order to characterise their swelling behaviour. This is demonstrated in Fig. 1B, where we see that while the temperature-induced deswelling of Hm and Dm particles exhibits an isotope effect, for both particles it is almost identical for different solvents.

**Table 1:** Summary of the properties of the microgels used for the SANS and SAXS experiments: Dm and Hm refer to the deuterated and hydrogenated particles, respectively.

Batch	*R*_*h*_(15 °C)[*nm*]	*R*_*h*_(27 °C)[*nm*]	[*R*_*h*_(15° C)/*R*_*h*_(45 °C)]^3^
Hm	115 ± 2	90 ± 2	17.9
Dm	125 ± 2	101 ± 2	21.4

*R*_*h*_[15 °C] and *R*_*h*_[27 °C] are the hydrodynamic radii measured with DLS at a temperature of 15 °C and 27 °C. Also shown is the swelling ratio defined as (*R*_*h*_[15 °C]/*R*_*h*_[45 °C])^3^.

The individual microgels at dilute conditions were also analysed using SANS, and the resulting particle dimensions were consistent with the DLS results (see SI and Fig. S2 for details). The conversion factor from weight fraction *C* to effective volume fraction *ϕ*_*eff*_, using *ϕ*_*eff*_ = *kC*, where *k* is the conversion or shift factor, was established by viscometry in the *ϕ* range 0.01–0.1, and by applying the Batchelor equation for effective hard spheres as described in detail in the methods section (see also SI and Fig. S1). The resulting values are summarised in Table 4. Note that this approach yields an effective volume fraction that is directly proportional to the number density *n*_*p*_. For conditions where the particle size exhibits strong deswelling, this *ϕ*_*eff*_ would then deviate from the “true” volume fraction given by *ϕ*_*eff*_ = *n*_*p*_ *V*_*p*_, where *V*_*p*_ is the volume of a single particle given by *V*_*p*_ = (4*π*/3)*R* ^3^, with *R* the radius of the microgel at this concentration. We have chosen this definition for *ϕ*_*eff*_ as it has become the standard approach in the microgel literature, but it is important to realise that these values cannot be used directly when attempting to compare experimental data on structure factors or pair distribution functions with computer simulations or theory for interacting colloids without taking into account the effective size of the particles at arbitrary concentrations.

**Table 4:** Summary of the results from capillary viscometry of the different particles and the resulting shift factors *k* used to determine the effective volume fraction *ϕ*_*eff*_ through *ϕ*_*eff*_ = *kC*, *C* is the microgel weight fraction.

Batch	*k*[15 °C]	*k*[27 °C]
Hm	18.1	12.4
Dm	15.5	11.6
Hm and Dm ZAC mixture	16.8	12.0

### Zero average contrast investigation

SANS experiments under zero average contrast conditions rely on a 50–50 mixture of deuterated and hydrogenated particles in a solvent with a scattering length density *ρ*_*s*_ that corresponds to the average of the values for the hydrogenated (*ρ*_*Hm*_) and deuterated (*ρ*_*Dm*_) particles. We have thus performed a systematic contrast variation for both particle types using SANS. Measurements were done for two different temperatures of *T* = 16.4 and 27.1 °C, and zero average contrast is achieved at a solvent mixture of 47%*v*/*v* H_2_O and 53% *v*/*v* D_2_O, independent of temperature. The results are summarised in Fig. S3 of the SI.

**Figure 2:**
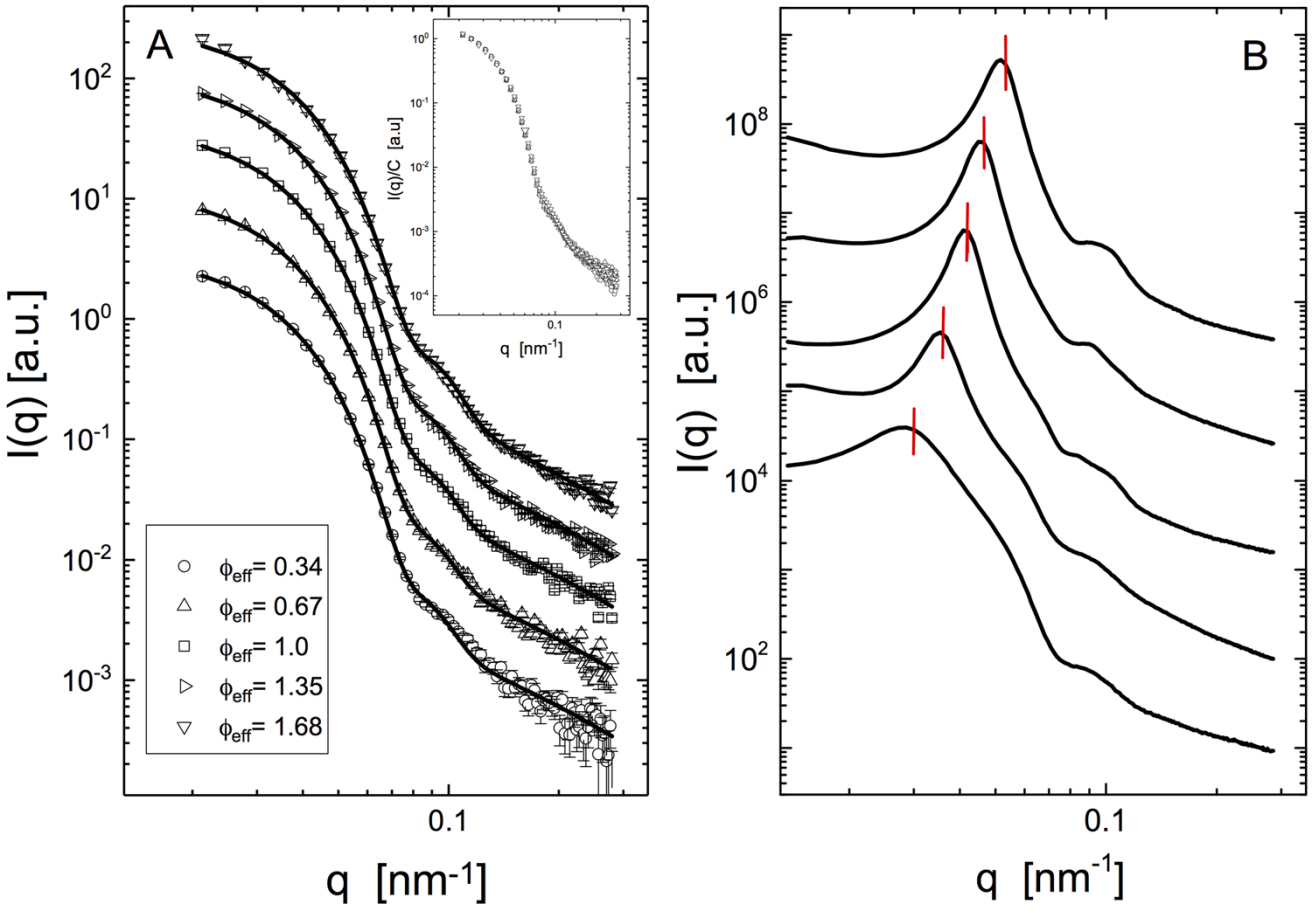
Small-angle neutron and X-ray scattering experiments. Shown are examples of the scattered intensity *I*(*q*) as a function of the scattering vector *q* for a 50–50 mixture of Hm and Dm particles at *T* = 16.4 °C in a ZAC solvent mixture for different values of the effective volume fraction 0.34 ≤ *ϕ*_*eff*_ ≤ 1.68 (**A**). Note that the data are in absolute units (cm^−1^), but the individual scattering curves are shifted in order to improve their visibility (shift factors: 1, 1.8, 4, 8 and 18 for increasing concentrations). The solid lines are the fits of the form factor for fuzzy spheres (see SI for details) to the experimental data. Also shown is the normalised scattering intensity $I(q)/C \sim \frac{d\sigma }{d{\rm{\Omega }}}(q)$ as a function of the scattering vector *q* for the same samples (inset), where *C* is the weight concentration. Examples of the scattered intensity *I*(*q*) as a function of the scattering vector *q* from SAXS experiments with a 50–50 mixture of Hm and Dm particles at *T* = 15 °C in a ZAC solvent mixture for different values of the effective volume fraction 0.34 ≤ *ϕ*_*eff*_ ≤ 1.68, bottom to top (**B**). The position *q* ^*^ of the maximum of the resulting structure factor peak is also indicated. The data is offset along the ordinate for clarity.

This solvent composition was then used to experimentally determine the particle form factor *P*(*q*) as a function of *ϕ*_*eff*_ using SANS on 50–50 mixtures of Hm and Dm particles. These mixtures were prepared based on the known molecular weight of the particles determined by static light scattering, which allowed us to calculate the number density *n*_*p*_ of the individual stock solutions. Examples for the resulting *q*-dependence of *dσ*(*q*)/*d*Ω at different packing fractions 0.34 ≤ *ϕ*_*eff*_ ≤ 1.68 (i.e., far beyond random close packing *ϕ*_*cp*_) at *T* = 16.4 °C are shown in Fig. 2. When normalised with the particle concentration, the individual data sets appear to overlap almost completely, already indicating that the effect of packing density on the particle shape is almost negligible even at the highest value of *ϕ*_*eff*_ = 1.68. The results from these measurements are also summarized in Tables 2 (for 15 °C) and 3 (for 27 °C) in the methods section. In order to verify these results, we also performed additional experiments using a small number of Hm particles as tracer particles that were then added to a majority phase of Dm particles under solvent conditions where the scattering contrast of the Dm particles was completely matched, i.e. where the SANS experiment provides the form factor of the Hm particles only. These measurements are summarised in Table S1 and Fig. S7 in SI and confirm the ZAC results, clearly indicating that a larger deswelling of the microgels only occurs at values of *ϕ*_*eff*_ ≫ *ϕ*_*cp*_.

The difference between the SANS-ZAC results and the SAXS data obtained with the same samples and shown in Fig. 2 is striking. The SAXS data at high *ϕ*_*eff*_ values are dominated by the contributions from the structure factor, while these are completely absent for the SANS data, and we measure contributions from *P*(*q*) only. We observe that a well-defined correlation peak typical for repulsive particles forms at high densities. Both the peak height and the position *q* ^*^ evolve with increasing *ϕ*_*eff*_, indicating that the characteristic center-center distance between particles *a*_*s*_ ~ 2*π*/*q* ^*^ strongly decreases with increasing packing fraction.

## Discussion

**Figure 3:**
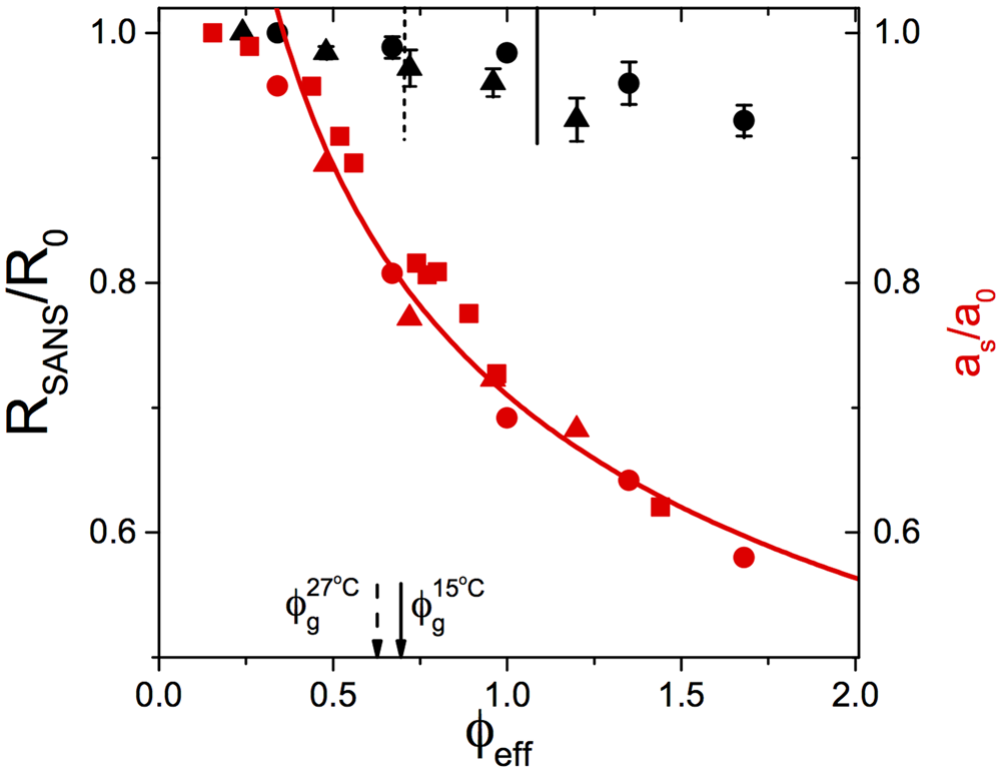
Summary of the results obtained by SANS and SAXS. Shown are: Normalised overall dimensions *R*_*SANS*_/*R*_*SANS*,0_ as a function of *ϕ*_*eff*_ obtained from SANS measurements using ZAC conditions at *T* = 15 °C (ILL) and 16.4 °C (PSI), respectively, where *R*_*SANS*,0_ corresponds to the value at *ϕ*_*eff*_ → 0 (solid black circles). The values correspond to the average obtained in different independent measurement campaigns at two different neutron sources, and the error bars reflect the standard deviations of these experiments. Also shown are the overall dimensions *R*_*SANS*_/*R*_*SANS*,0_ as a function of *ϕ*_*eff*_ at *T* = 27.1 °C (ILL) and 27.4 °C (PSI) (solid black triangles). The average normalised center-center distances *a*_*s*_/*a*_0_ from SAXS are shown as the red solid circles for *T* = 15 °C and triangles for *T* = 27 °C, where *a*_0_ = 2*R*_*h*_[*T*] × *R*_*SANS*_/*R*_*SANS*,0_. The average normalised center-center distances *a*_*s*_/*a*_0_ from CLSM are shown as the red solid squares for *T* = 15 °C. The red solid line represents the dependence of the normalised center-center distance on the number density for particles interacting via a soft repulsive potential given by ${a}_{s}/{a}_{0} \sim {\varphi }_{eff}^{-1/3}$ expected for *ϕ*_*eff*_  ≥ *ϕ*_*rcp*_, where *ϕ*_*rcp*_ corresponds to random close packing. The location of the glass transition is indicated by the black solid arrow (*T* = 15 °C) and the black dashed arrow (*T* = 27 °C) at the bottom, and an estimate of the volume fraction *ϕ*_*eff*_ where the outer shell touches the dense core of the nearest neighbour particles is given as the short solid black (*T* = 15 °C) and dashed (*T* = 27 °C) lines at the top.

A comparison between the graphs in Fig. 2 reveals a surprising behavior. While the increasing number density of particles results in the expected decrease of the average center-center distance *a*_*s*_ between particles, this seems to have a very small effect on the particle size and shape only. We can quantify this by analysing all SANS data using the fuzzy sphere model (see SI for details)^12^. The experimental data is very well reproduced by this model (see Fig. 2), and the concentration dependence of the overall particle size given by *R*_*SANS*_ is summarised in Fig. 3. Plotted is a normalised value *R*_*SANS*_/*R*_*SANS*,0_, where *R*_*SANS*,0_ corresponds to the value at *ϕ*_*eff*_ → 0 obtained in the form factor measurement, in order to facilitate comparisons between different measurements and temperatures. The overall particle size remains remarkably constant with increasing *ϕ*_*eff*_, and only decreases appreciably for *ϕ*_*eff*_ ≳ 1.0, i.e. far above random close packing.

At the same time the average normalised distance *a*_*s*_/*a*_0_ between particles as obtained from the position of the correlation peak *q* ^*^ in the SAXS data, where we follow ref. 16 and use *a*_*s*_ = 2.2*π*/*q* ^*^, decreases strongly for *ϕ*_*eff*_ ≳ 0.4. For the normalisation we use the intensity-weighted particle diameter *a*_0_ = 2*R*_*h*_[*T*] × *R*_*SANS*_/*R*_*SANS*,0_ given by the measured hydrodynamic radius for this temperature *R*_*h*_[*T*] and corrected for the concentration-dependent deswelling as measured in the SANS-ZAC experiment. It is interesting to note that it follows a relationship *a*_*s*_/*a*_0_ ~ *ϕ*_*eff*_^−1/3^, i.e. it appears to depend on the particle number density only, and thus seems to correspond to an average nearest neighbor distance between particles that uniformly occupy the available space. Similar behavior is known to occur in charged colloids interacting via a long range repulsive Yukawa potential for volume fractions where the long repulsive tails of the potentials significantly overlap^16^. This is in agreement with the fact that PNIPAm microgels are believed to interact with a very soft repulsive potential given for example by a so-called Hertzian potential^17^. We can in fact directly observe the decrease of the average particle distance with increasing concentration by extending our investigations to particle sizes with radii of several hundred nanometer that can directly be followed using confocal laser scanning microscopy. Here we use particles with a hydrodynamic radius of about 375 nm at *T* = 15 °C that have previously been employed to study interactions and phase behaviour of soft particles^17, 18^. Using the CLSM allows us to directly determine the average interparticle distance *a*_*s*_. When normalised with the corresponding value *a*_0_ = 0.748 *μ*m, the resulting values of *a*_*s*_/*a*_0_ perfectly overlap with those obtained with SAXS for the smaller particles in the ZAC mixture (Fig. 3). The available data for swollen microgels at *T* = 15 °C shown in Fig. 3 is clearly not compatible with an often proposed scenario where PNIPAm particles adapt their size once they reach close contact. Here the overall size *R*_*SANS*_ and the center-center distance *a*_*s*_ would decrease in a similar fashion for sufficiently high volume fractions. Instead, our data strongly suggests that the microgel particles initially interpenetrate up to values of *ϕ*_*eff*_ far above random close packing. When considering the internal core-shell structure of the particles with a dense (and densely cross-linked) core and a much looser shell, it is tempting to assume that a more significant decrease of the overall size only occurs once the outer shells are fully interpenetrated and start to touch the inner core. For the given values of the core radius *R*, the shell thickness 2*σ*_*shell*_ and the known number density *n*_*p*_, we can estimate the effective volume fraction where this should happen. The corresponding value is indicated in Fig. 3, and indeed appears to mark the point where the overall size starts to decrease more strongly with increasing *ϕ*_*eff*_.

A test of this hypothesis can be done by looking at the effect of temperature. We have performed a complete set of SANS and SAXS measurements also at *T* = 27 °C, i.e. under conditions where the particles partially de-swell but are still repulsive (see Figs S4 and S5 in SI for the corresponding scattering data). Under these conditions we expect the particles to experience a slightly harder repulsive potential. Together with the fact that initially the outer shell appears to de-swell more than the core, we then expect to see a narrower region where particles are able to interpenetrate only, and a decrease of the value of *ϕ*_*eff*_ where the outer shell of a particle touches the core of its nearest neighbours to approximately *ϕ*_*eff*_ ≈ 0.7. Figure 3 demonstrates that this is indeed the case and that *R*_*SANS*_/*R*_*SANS*,0_ decreases appreciably already for *ϕ*_*eff*_ ≳ 0.7.

**Figure 4:**
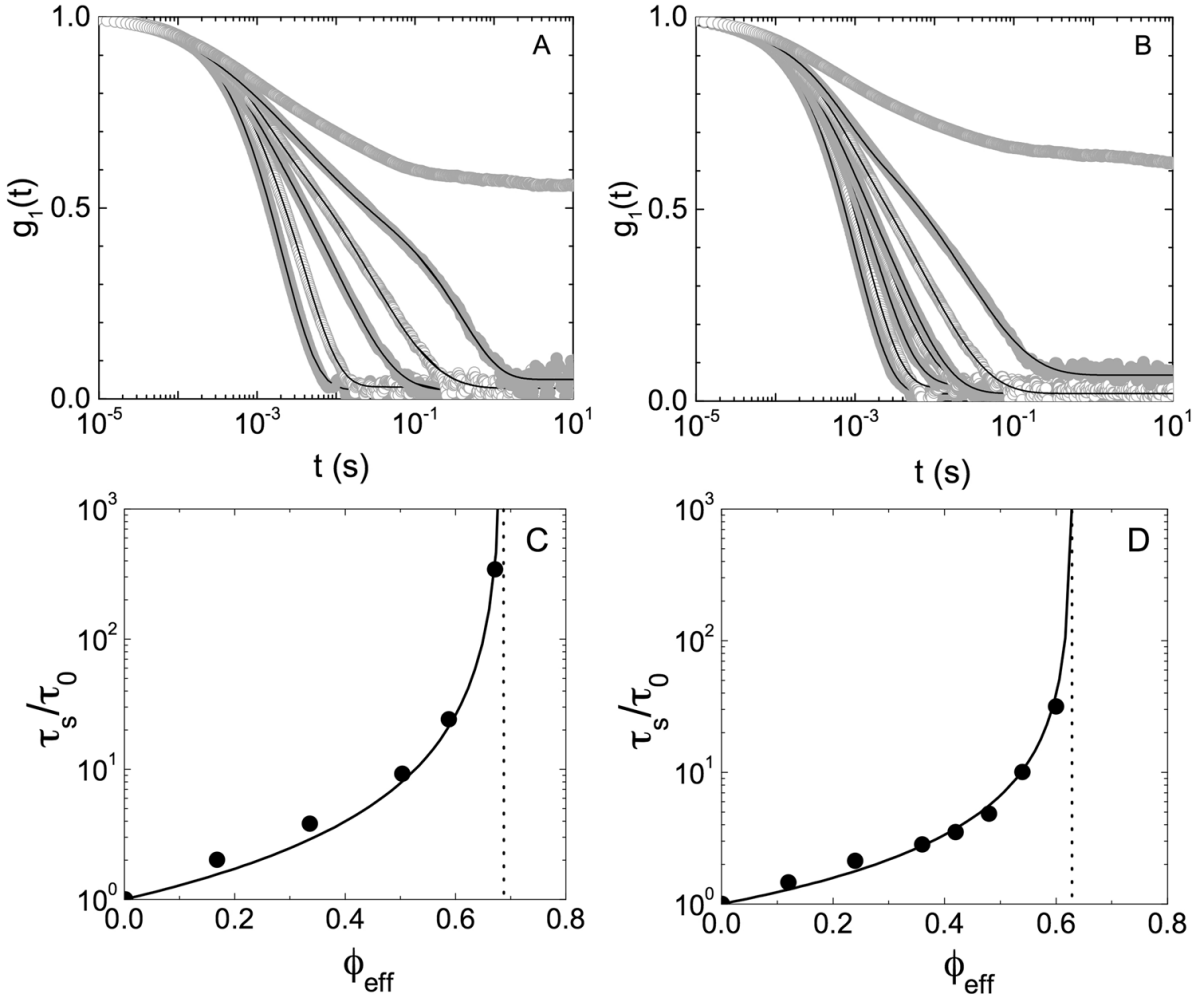
Dynamics of dense suspensions. (**A**) Normalized field autocorrelation functions *g*_1_(*t*) as a function of delay time *t* for 50–50 particle mixtures in ZAC solvent at *T* = 15 °C and effective volume fractions *ϕ*_*eff*_ = 0.17, 0.34, 0.50, 0.59, 0.67, 0.84, from left to right. (**B**) Normalized field autocorrelation functions *g*_1_(*t*) as a function of delay time *t* for 50–50 particle mixtures in ZAC solvent at *T* = 27 °C and effective volume fractions *ϕ*_*eff*_ = 0.12, 0.24, 0.36, 0.42, 0.48, 0.54, 0.6, 0.96, from left to right. (**C**) Normalized slow relaxation time *τ*_*s*_/*τ*_0_ as a function of *ϕ*_*eff*_ at *T* = 15 °C. (**D**) Normalized slow relaxation time *τ*_*s*_/*τ*_0_ as a function of *ϕ*_*eff*_ at *T* = 27 °C. Also shown are fitted curves *τ*_*s*_/*τ*_0_ = (1 − *ϕ*_*eff*_/*ϕ*_*g*_)^−*α*^ as solid lines, where *α* = 1.6 for *T* = 15 °C and *α* = 1.2 for *T* = 27 °C, and the resulting estimate of the glass transition volume fraction *ϕ*_*g*_ as the dotted lines.

While the data shown in Fig. 3 are incompatible with a model where individual particles compress upon close packing, such an interpretation strongly relies on our estimate of the effective volume fraction *ϕ*_*eff*_ using viscometry data as described in the Methods section (equation (4)). It is well-established that PNIPAm particles with such a polydispersity exhibit a glass transition at *ϕ*_*g*_ ≳ 0.6, i.e. at somewhat higher volume fractions than an equivalent hard sphere system^3, 18^. Moreover, we expect that *ϕ*_*g*_ increases with increasing particle softness. We have thus also investigated the existence and location of a glass transition using DLS. Examples for the concentration dependence of the corresponding field autocorrelation functions *g*_1_(*t*) are shown in Fig. 4 for a 50–50 mixture of particles in the ZAC solvent at *T* = 15 °C and 27 °C, respectively. These correlation functions exhibit the characteristic slowing down and the formation of a second decay of soft particles in an ergodic fluid state approaching a glass transition with increasing *ϕ*_*eff*_ for *ϕ*_*eff*_ ≦ 0.67 for *T* = 15 °C and for *ϕ*_*eff*_ ≦ 0.60 for *T* = 27 °C.

We analyse the slow decay that arises due to the influence of the cages formed by the neighboring particles using a combination of two decay processes following

3 $${g}_{1}(t)={A}_{f}\exp (-\,t/{\tau }_{f})+{A}_{s}\exp [-\,{(t/{\tau }_{s})}^{\beta }]$$

where *A*_*f*_ and *A*_*s*_ are the amplitudes of the fast and slow processes, *τ*_*f*_ and *τ*_*s*_ their relaxation times, and *β* is a stretching exponent related to the slow or alpha relaxation process. The correlation functions are well described by eqn. (3), with values of *β* ~ 0.5–0.8. We observe a dramatic increase of *τ*_*s*_ with increasing *ϕ*_*eff*_ given by *τ*_*s*_(*ϕ*_*eff*_)/*τ*_0_ = (1 − *ϕ*_*eff*_/*ϕ*_*g*_)^−*α*^, where *τ*_0_ is the characteristic decay time obtained for a very dilute non-interacting sample at *ϕ*_*eff*_ = 1.2 × 10^−3^. The resulting *ϕ*_*eff*_-dependence of *τ*_*s*_ for *T* = 15 °C and 27 °C is shown in Fig. 4, and indicates that a glass transition occurs at values of *ϕ*_*g*_ ≃ 0.69 for *T* = 15 °C and *ϕ*_*g*_ ≃ 0.63 for *T* = 27 °C. DLS measurements performed with higher concentrations indeed clearly show that the suspensions then are in a non-ergodic glassy state, where we observe large variations of the amplitude of the slow process for different measurements, and large variations between measurements without and with a slow rotation of the cell used to create an ergodic average for glassy samples^19^. These values of *ϕ*_*g*_ are in good agreement with expectations for particles of this cross-link density and softness^11^, and reflect the fact that PNIPAm particles become less soft upon deswelling at higher temperatures. These findings provide strong support for the determination of *ϕ*_*eff*_ using eqn. (4) and the values of *k* given in Table 4. They thus confirm that the PNIPAm particles studied primarily interpenetrate at concentrations above close packing, and show only a small amount of compression even at effective volume fractions *ϕ*_*eff*_ as high as 1.68.

**Table 2:** Summary of the properties of the Hm-Dm mixture of microgels obtained from SANS experiments under ZAC conditions at all concentrations investigated.

*C* [%*wt*]	*ϕ*_*eff*_	*R*_*SANS*_ [*nm*]	2*σ*_*shell*_ [*nm*]	2*σ*_*shell*_/*R*_*SANS*_	*PD*
2	0.34	94.6 ± 0.3	35.8 ± 0.2	0.38	0.15 ± 0.02
4	0.67	92.7 ± 0.3	33.2 ± 0.2	0.36	0.13 ± 0.02
6	1.0	92.7 ± 0.3	32.8 ± 0.2	0.35	0.13 ± 0.02
8	1.35	89.2 ± 0.3	29.4 ± 0.2	0.33	0.15 ± 0.02
10	1.68	86.8 ± 0.3	28.4 ± 0.2	0.33	0.15 ± 0.02

Shown are the number-averaged values of the overall radius *R*_*SANS*_ = *R* + 2*σ*_*shell*_, the fuzzy shell 2*σ*_*shell*_, the size ratio 2*σ*_*shell*_/*R*_*SANS*_, and the polydispersity *PD* as obtained from a fit of the fuzzy sphere model to the experimental data for *T* = 15 °C.

While we can thus exclude the previously postulated view that particles start to de-swell at and above close packing and adapt their size accordingly^9, 11^, there still remains the possibility that they also change their shape and exhibit a facetted structure similar to dense emulsions or foams as described in a recent publication by Cloitre and collaborators^10, 20^. We thus need to investigate whether the fact that the experimentally determined particle form factor changes weakly only (Fig. 2) is compatible with a change in particle shape from a fuzzy core-shell sphere to a densely packed space-filling facetted structure. This is directly related to the question on how discriminative different structural models are when compared to the limited resolution of a SANS experiment for a given instrumental resolution function.

We address this question by comparing the concentration-induced changes in the scattering data with those that would be expected from a small change in the measured effective particle polydispersity. Here we do not use an approach where we compare different explicit geometrical models, but rather use the fact that weak shape anisotropy in dispersions of particles without preferential alignment results in azimuthally averaged scattering data that cannot be distinguished from those obtained with polydisperse spherical particles. Starting point is the observation that we have not seen any increase of the resulting effective polydispersity in the analysis of the experimental SANS data at concentrations above *ϕ*_*cp*_. However, we then need to verify whether we would be able to see a small change in polydispersity that may occur as a result of a shape change.

We use a so-called residual plot [*I*(*ϕ*_*eff*,2_)/*C* − *I*(*ϕ*_*eff*,1_)/*C*]/*I*(*ϕ*_*eff*,1_/*C*) to address this point, where *C* is the corresponding weight concentration, to highlight differences between the concentration-normalized scattering data *I*(*ϕ*_*eff*_)/*C* obtained at two different volume fractions *ϕ*_*eff*,1_ and *ϕ*_*eff*,2_, respectively. We focus in particular on *q*-values around *q* ≈ 0.07 nm^−1^, where the first minimum in the form factor appears. We then compare this data to the changes in the theoretical form factor *P*_*fit*_(*q*) for a given *ϕ*_*eff*,2_ caused by an increase of the effective polydispersity from 12 to 17% and 12 to 22%, respectively. An effective polydispersity of 12% was chosen as the starting value as this is needed to reproduce the measured form factors within the fuzzy sphere model due to the contributions of both the size polydispersity of the individual particles (Hm and Dm) as well as the small size difference between them. The data are again represented in the form of a residual plot given by [*P*_*fit*_(*ϕ*_*eff*,2_, *PD*_2_) − *P*_*fit*_(*ϕ*_*eff*,1_, *PD*_1_)]/*P*_*fit*_(*ϕ*_*eff*,1_, *PD*_1_). Here [*P*_*fit*_(*ϕ*_*eff*,2_, *PD*) is the theoretical form factor of the fuzzy sphere model at a volume fraction *ϕ*_*eff*,2_ and for a polydispersity *PD*, where *PD*_1_ = 12% and *PD*_2_ = 17% or 22%, respectively. The values for the increase in polydispersity due to shape anisotropy were chosen based on a number of different geometrical shapes with equal volume compared to the corresponding sphere such as for example an icosidodecahedron, which gives a ratio of 1.07 when one follows the sharp edges.

**Figure 5:**
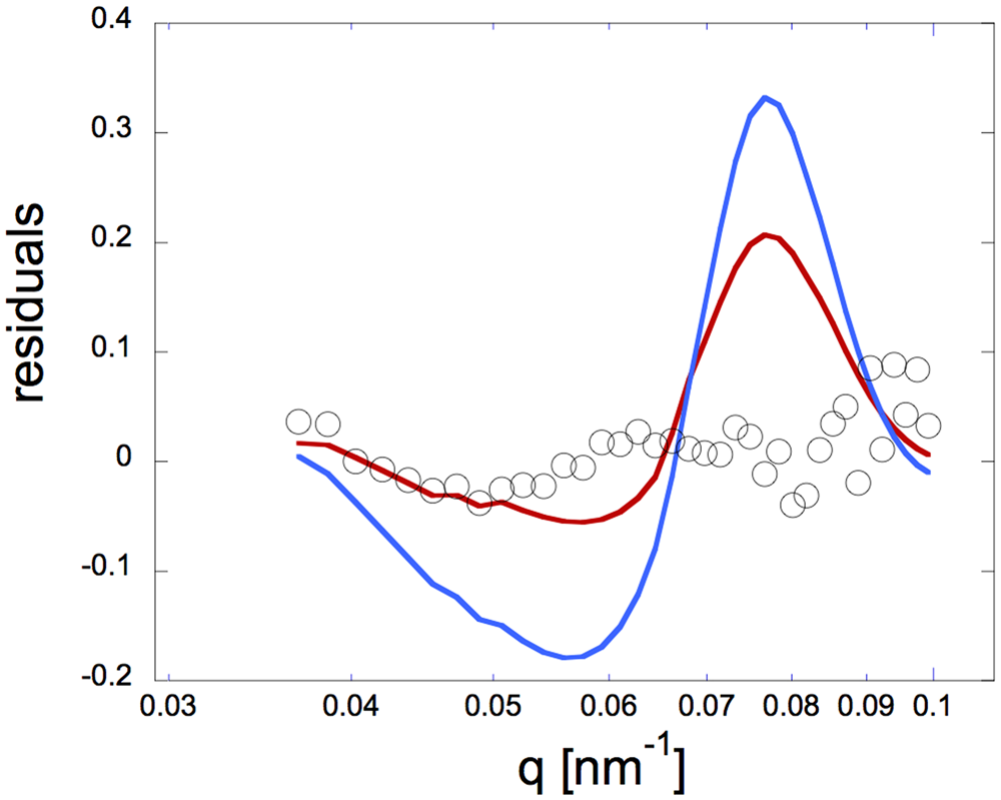
Assessing the sensitivity of the SANS analysis. Comparison of the concentration-normalized scattered SANS intensity *I*(*q*)/*C*, where *C* is the weight concentration, in ZAC contrast from samples with an effective volume fraction *ϕ*_*eff*_ = 0.67 and *ϕ*_*eff*_ = 1.0 in a residual plot [*I*(*ϕ*_*eff*_ = 1.0)/*C* − *I*(*ϕ*_*eff*_ = 0.67)/*C*]/*I*(*ϕ*_*eff*_ = 0.67/*C*). Also shown are calculated form factors for different values of the effective polydispersity also as residual plots given by [*P*_*fit*_(*ϕ*_*eff*,2_, *PD*_2_) − *P*_*fit*_(*ϕ*_*eff*,1_, *PD*_1_)]/*P*_*fit*_(*ϕ*_*eff*,1_, *PD*_1_), where *PD*_1_ = 12% and *PD*_2_ = 17% (red line) or 22% (blue line), respectively.

As shown in Fig. 5, such a residual plot is indeed capable of visualising the small characteristic change in *R*_*SANS*_/*R*_*SANS*,0_ of around 1% for an increase in volume fraction from *ϕ*_*eff*_ = 0.67 to *ϕ*_*eff*_ = 1.0. Note that for the same increase in *ϕ*_*eff*_ the normalised interparticle distance *a*_*s*_/*a*_0_ decreases by about 14%. However, the figure also demonstrates that already a small change of the effective polydispersity of 5%, i.e. from 12 to 17%, results in a drastic change of *P*_*fit*_(*q*) around *q* ≈ 0.07 nm^−1^ that is clearly outside the statistical noise of the data and results in systematic deviations when compared to the changes induced by the increasing concentration. The figure also illustrates that despite the instrumental smearing in a SANS experiment the obtained scattering data is highly sensitive to small variations in the fit parameters, thus underlining the small statistical errors estimated for the characteristic properties of the fuzzy sphere model determined in the analysis of the SANS data under ZAC conditions. In this context it is however also important to point out that when using the fuzzy sphere model to analyse SANS or SAXS data for microgels, one generally notes a small but systematic difference between the results from SANS/SAXS (*R*_*SANS*_) and DLS (*R*_*h*_). It has already been pointed out by Stieger *et al*.^12^ in their original description of the fuzzy sphere model that this difference is likely caused by a few dangling polymer chains attached to the particle surface that contribute to the hydrodynamics, which have a concentration that is not high enough to be detected by SANS. As these dangling chains cannot be seen by SANS, we can also not exclude that they deswell at high packing fractions. However, our main conclusion is drawn from the comparison of the average center-center distance *a*_*s*_ between neighbouring particles and the measured size *a*_0_ = 2*R*_*SANS*_. Even if these invisible dangling chains would deswell at large values of *ϕ*_*eff*_, the visible part of the outer shells still must interpenetrate since we find *a*_*s*_ ≪ *a*_0_ under these conditions.

Our results first seem at odds with another recent study by Gasser *et al*. using the same technique^21^. In their publication the authors concluded that microgels would shrink for *ϕ*_*eff*_ ≳ 1.0 with very little interpenetration, i.e. they found that the overall microgel size would closely follow a relationship *R*_*SANS*_/*R*_*SANS*,0_ ~ *ϕ*_*eff*_^−1/3^ for *ϕ*_*eff*_ ≳ 1.0. Similar results were also recently described by Scotti *et al*. from tracer studies^22^, where SANS contrast matching has been used to investigate the concentration dependence of protonated larger (or smaller) particles in a matrix of contrast-matched smaller (larger) deuterated particles. An analysis using the fuzzy sphere model, i.e. analogous to the one used by us, has shown clear signs of deswelling for *ϕ*_*eff*_ ≳ 1.0. However, it is important to carefully look at the different particle systems studied in refs 21, 22 and in our own work. The microgels used by Scotti *et al*. and Gasser *et al*. possess a smaller degree of cross-linking (2%), and thus are much softer as indicated by a larger swelling ratio and shift factor *k* as well as a much larger size and softness heterogeneity for the mixtures. In the case of the study by Gasser *et al*., the larger softness also results in the absence of a fluid-solid transition up to the highest packing fractions investigated. It appears that particle softness not only strongly influences the macroscopic flow properties as already described in some detail in the past^23^, but also the response of individual particles to dense packing.

When looking at the results described by Scotti *et al*., another possible response of soft microgels could also occur for mixtures differing in their elasticity. Here the softer component could show preferential de-swelling at large packing fractions above close packing. As our particles indeed show a small difference in softness as expressed for example through the shift factor *k*, we have also investigated the behavior of the softer Hm particles separately by performing tracer measurements, where a small number of Hm particles was added to a majority phase of Dm particles under solvent conditions where the Dm particles were completely matched, i.e. where the SANS experiment again provided the form factor of the Hm particles only. No difference for the deswelling behavior obtained in the tracer study (Hm particles only) and the ZAC measurement (average form factor for Hm and Dm) was observed, as shown in SI (Fig. S7), thus confirming our previous interpretation.

**Figure 6:**
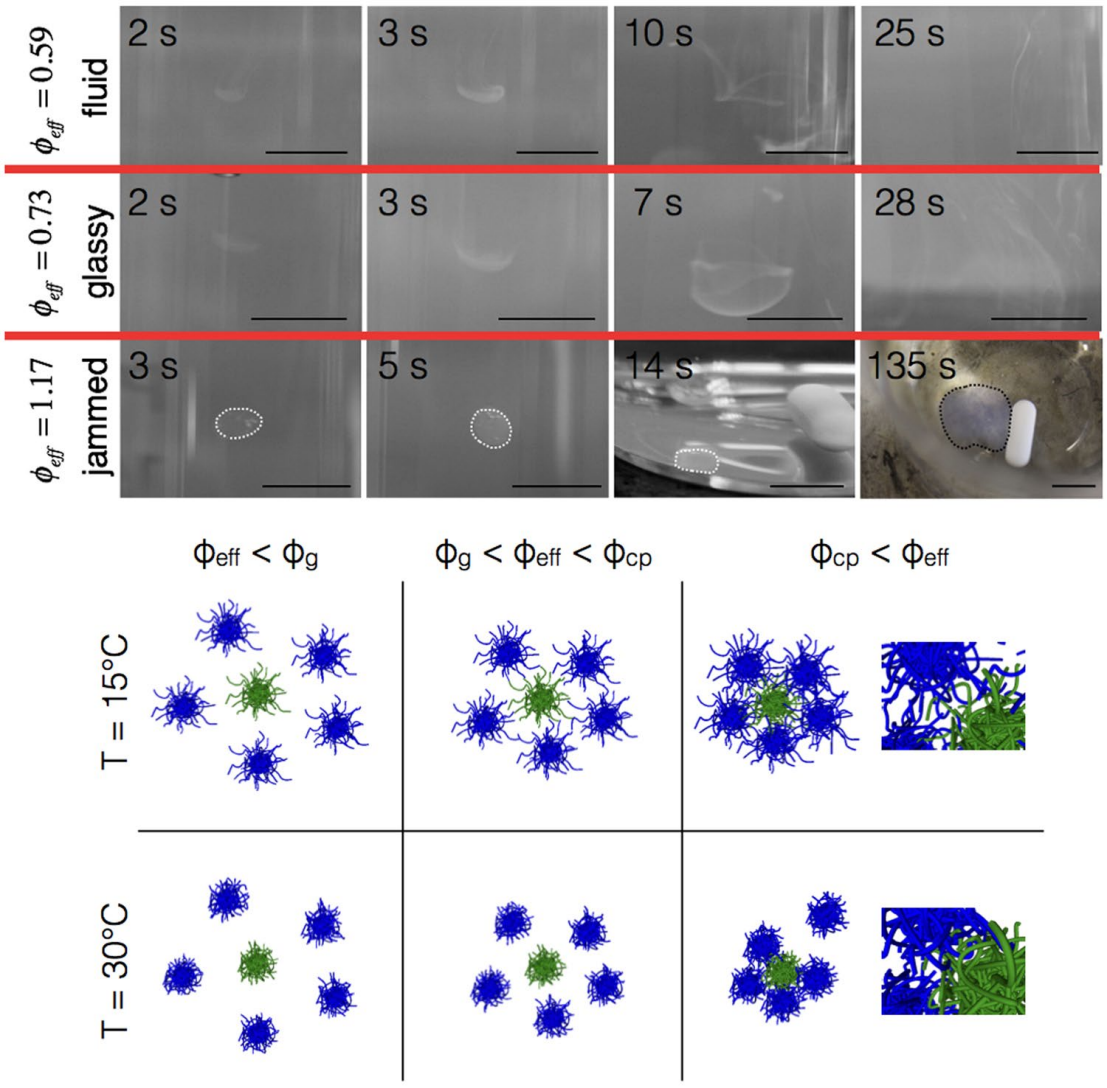
Slow dissolution of jammed samples. Upper panel: Dissolution of a small droplet of a microgel sample equilibrated at *T* = 15 °C upon injection into a water bath at *T* = 30 °C. Top row: Initial sample concentration *ϕ*_*eff*_ = 0.59. Middle row: Initial sample concentration *ϕ*_*eff*_ = 0.73. Bottom row: Initial sample concentration *ϕ*_*eff*_ = 1.17. Pictures shown were taken at different times after injecting the droplet. Scale bars correspond to 10 mm. Lower panel: Schematic description of the response of a microgel dispersion to a rapid temperature jump from 15 °C (*T* ≪ *T*_*VPT*_) to 30 °C (*T* ≲ *T*_*VPT*_) at three different volume fractions *ϕ*_*eff*_ < *ϕ*_*g*_ (left), *ϕ*_*g*_ < *ϕ*_*eff*_ < *ϕ*_*cp*_ (middle), and *ϕ*_*eff*_ > *ϕ*_*cp*_ (right), respectively, where *ϕ*_*cp*_ stands for the volume fraction where particles are close packed or jammed and start to significantly interpenetrate. Also shown is a magnified view of the temporary entanglements at *ϕ*_*eff*_ > *ϕ*_*cp*_ (right), which become buried and trapped after the temperature jump.

Our results thus clearly indicate a significant degree of interpenetration of the microgels at *ϕ*_*eff*_ ≳ *ϕ*_*cp*_. There is in fact evidence to believe that interpenetration of microgels results in a modified effective pair potential with an additional attractive component setting in once the outer shells interpenetrate. This results in much slower relaxation of structures formed at low temperatures and high densities upon a sudden increase of the temperature, in addition to the slowing down caused by the entanglement of the chains in the interpenetrated regions. This can even be seen in macroscopic observations as shown in Fig. 6. Here we show the dissolution of a small drop of about 20 *μ*l (corresponding to a droplet radius of approximately 1.5 mm) of a microgel sample equilibrated at a temperature of *T* = 15 °C, injected into a large volume of water at *T* = 30 °C. The figure shows a series of pictures taken at different times after injection, and for 3 different initial concentrations that correspond to a sample state in either the fluid or the glassy regime, respectively. As shown in Fig. 6 top row, for samples in the fluid state (*ϕ*_*eff*_ = 0.59 < *ϕ*_*g*_ ≃ 0.69) the droplet dissolves within a few seconds. The same behavior is observed for a glassy sample just beyond the glass transition (*ϕ*_*eff*_ = 0.73 > *ϕ*_*g*_), i.e. where caging and weak interpenetration are the dominant factors determining the solid-like properties of the sample (Fig. 6 second row). However, when injecting a sample at high *ϕ*_*eff*_ = 1.17, i.e. from a strongly interpenetrated and weakly compressed state, the droplet initially only swells weakly, changes turbidity as a result of the increased temperature, sediments to the bottom of the flask, continues to swell and spread considerably, but remains intact for several minutes (Fig. 6 third row). This is very different from the behaviour one would expect for close-packed compressed particles that partly collapse as a result of the temperature increase, and demonstrates the importance of interpenetration for the resulting dynamic properties of the samples.

These phenomenological observations allow us to sketch a simple and schematic summary of the local structure of our microgel suspensions at different densities, and their response to a sudden change in temperature. This is illustrated in the bottom panel of Fig. 6, where we show the initial arrangement of particles at *ϕ*_*eff*_ < *ϕ*_*g*_, *ϕ*_*g*_ < *ϕ*_*eff*_ < *ϕ*_*cp*_, and *ϕ*_*eff*_ > *ϕ*_*cp*_, respectively, and their response to a sudden increase of the temperature as experienced in the course of the experiment described above. Here *ϕ*_*cp*_ stands for the volume fraction where particles are close packed or jammed and start to significantly interpenetrate. For a fluid suspension at *ϕ*_*eff*_  < *ϕ*_*g*_, the temperature increase results in a fast decrease of *ϕ*_*eff*_ in the injected droplet. As the increased temperature is still below the transition temperature *T*_*VPT*_, the particles are interacting via a purely repulsive potential, and the decrease in *ϕ*_*eff*_ has little consequences on the dissolution of the injected droplet. For a glassy suspension at *ϕ*_*g*_ < *ϕ*_*efff*_ < *ϕ*_*cp*_, the temperature increase again results in a fast decrease of *ϕ*_*eff*_, effectively melting the glass, and thus resulting in a faster dissolution of the injected drop as the long time collective diffusion coefficient of the particles in the drop increases strongly. For *ϕ*_*eff*_ > *ϕ*_*cp*_, however, the scenario encountered changes completely. As the weakly cross-linked outer shells of the particles now interpenetrate with those of their nearest neighbours, leading to the formation of chain entanglements between chains on different particles, these entanglements act as transient bonds between neighbouring particles. A rapid temperature increase thus again results in a reduction of the particle size, but as some particles are connected through temporary entanglements, this leads to temporary cluster and network formation. In the interpenetrated and temporarily cross-linked outer shells, this results in a further increase of the local polymer concentration, thus further enhancing the formation of entanglements, and increasing the escape time from these partially buried temporary bonds between particles. As the injected drop is initially already in a jammed solid-like state, the temperature jump occurs fast enough to precede dissolution, and thus results in an enhanced temporary cross-link formation between neighbouring particles, resulting in a transient gel formation that resists dissolution. The injected drop thus swells initially, and dissolves only slowly through a slow release of chain entanglements in neighbouring particles. This response would be very different if the outer shells would not interpenetrate, but the particles had instead adapted their size through de-swelling in order to always maintain a condition of close packing only, i.e. with a particle diameter given by the center-center distance between nearest neighbours, *a*_0_ = *a*_*s*_, as postulated by a number of previous studies^9, 21^. In this case the increase in temperature that accompanies the injection would again result in a simple melting of the jammed particle glass, thus enhancing the dissolution. Based on these phenomenological observations we in fact expect that shell interpenetration and temporary entanglements not only influence the dissolution kinetics of the droplets in our simple experiment, but will have significant consequences in temperature-induced phase transition kinetics or flow behaviour for samples at densities above close packing.

## Conclusion

Our results clearly indicate that while thermoresponsive microgels indeed show a variety of fascinating structural and dynamic properties, care has to be taken when trying to use them as simple model systems to investigate phase transitions or look at various macroscopic viscoelastic properties. The individual particles appear to adjust to dense packing conditions differently and in a much more complex manner then previously assumed^9–11^. Their ability to interpenetrate and deswell appears to strongly depend on particle softness, internal cross-link density distribution and temperature, which will definitively require a second look at some of the previously postulated microscopic structural models used to describe flow properties of dense suspensions of microgels^10^. First of all, we expect that strongly interpenetrating outer shells of microgel particles will influence dynamic quantities due to the influence of chain entanglements, thus slowing down various relaxation processes. Moreover, as we expect that the effective interaction potential between microgels at high packing densities will strongly depend on whether particles interpenetrate or deswell, this also indicates that we can expect that a variation of temperature often used to change the effective volume fraction of microgels in order to drive the system across fluid-solid transitions and form either glasses or jammed particle systems will be accompanied by a corresponding change in the potential already before the volume phase transition temperature. This makes the interpretation of such experiments much less straightforward than often assumed^7, 9^. Given the increasing use of thermoresponsive microgels as model systems to study a broad range of phenomena as diverse as structural transitions in 2 and 3 dimensions^24, 25^, or colloidal filtration^26^, we also expect that the ability of microgels to respond to dense packing through interpenetration and/or deswelling will have important consequences for the interpretation of these investigations.

## Methods

### Particle synthesis

Hydrogenated (Hm) and deuterated (Dm) PNIPAm particles were synthesized using precipitation polymerization in the presence of a surfactant. Hm was synthesized in 95 g of water using N-N-isopropylacrylamide (1.82 g, Acros Organics) as the monomeric unit. N, N-methylenebis(acrylamide) (0.09 g, Sigma-Aldrich) was used as a cross-linker and sodium dodecyl sulfate (SDS, 0.041 g, Duchefa Biochemie) as the surfactant. Potassium persulfate (0.06 g dissolved in 5 g H_2_O, Sigma Aldrich) was used as the initiator. Dm was synthesized in 95 g H_2_O using a deuterated monomer, d _7_-N-isopropylacrylamide(1.99 g, Polymer Source), 0.145 g of cross-linker and 0.038 g of surfactant. Except for N-N-isopropylacrylamide, which was re-crystallized in hexane, all chemicals were used as received. The differences in the added amounts of cross-linker and surfactant were found necessary in order to match the particle softness and swelling behavior in the two batches. The reactions were carried out in a three-necked round bottom flask under a constant flow of argon at a constant stirring speed of 250 rpm. The temperature was stabilized at 70 °C before the injection of initiator took place. The suspensions were left for 6 h at 70 °C and thereafter left to cool down over night at room temperature. The suspensions were dialysed for 2 weeks against de-ionized water using a dialysis tube with molecular weight cut-off of 10000 (Spectrum laboratories) in order to remove excess monomers, oligomers and surfactant until the conductivity was found to be below 1 *μ*S/cm.

### Dynamic light scattering (DLS)

DLS experiments were performed on a commercial goniometer system (3D LS Spectrometer from LS Instruments, Switzerland) implementing the modulated 3D cross-correlation scheme to suppress contributions from multiple scattering^27, 28^. Measurements for the particle characterisation were done at different scattering angles (30° ≤ *θ* ≤ 130°). Three measurements were made at each angle and analysed using a first order cumulant analysis from the initial part of the correlation function in order to extract the decay constant Γ(*q*), where *q* = (4*πn*/*λ*_0_)*sin*(*θ*/2) is the magnitude of the scattering vector, *n* is the dispersion index of refraction, and *λ*_0_ = 632 nm is the vacuum wave length of the laser used. The final diffusion coefficients *D* and hydrodynamic radii *R*_*h*_ were then extracted using Γ = *Dq* ^2^. The resulting particle dimensions are summarised in Table 1.

### Small-angle neutron scattering (SANS)

Small-angle neutron scattering experiments were performed at the instrument D11 at the Institute Laue-Langevin (ILL), France. Two instrument settings were used to cover the needed *q*-range, resulting in sample-to-detector and collimation distances of 34 m and 8 m, respectively. A wavelength of 0.8 nm was used. Additional SANS experiments were performed at the instrument SANS-1 at the Swiss neutron source SINQ, Paul Scherrer Institut (PSI), Switzerland. Sample-to-detector and collimation distances of 18 m and a wavelength of 0.8 nm were used. The raw spectra were corrected for background from the solvent, sample cell, and electronic noise by conventional procedures. Furthermore, the two-dimensional isotropic scattering spectra were corrected for detector efficiency by dividing by the incoherent scattering spectra of pure water and azimuthally averaged. The results from these measurements are summarized in Tables 2 (15 °C) and 3 (27 °C), respectively.

For the contrast variation measurements used to determine the ZAC condition, 0.5 wt% samples of each set of particles were prepared in different H_2_O/D_2_O ratios. Measurements were performed using a sample-to-detector distance and collimation distance of 34 m at ILL and 18 m at PSI, and all data were corrected for background, solvent densities and the number density difference. The square root of the differential cross-section at *q* = 0 was plotted as a function of H_2_O content in the solvent for the two set of particles. The intersection of the two curves was then used to determine the ZAC solvent mixture. This was performed at temperatures of 16.4 °C (PSI) and 27.1 °C (ILL), respectively. For the final ZAC particle mixture, the differences in solvent densities and the number density difference between the two set of particles was also taken into account.

### Small-angle X-ray scattering (SAXS)

Small-angle x-ray scattering experiments were conducted at the cSAXS beam line at the Swiss Light Source SLS, Paul Scherrer Institute (Villigen, Switzerland). An X-ray beam with an energy of 11.2 keV was used, corresponding to a wavelength *λ* = 0.111 nm, with which a *q*-range of 0.014–1.5 nm^−1^ is covered. The measurements were performed using disposable 1.5 mm thickness quartz capillaries (Hilgenberg GmbH, Malsfeld, Germany), introduced in a metallic sample holder. The temperature was controlled, within ±0.4 °C, using an external recirculating water bath Table 3. The SAXS data was then analysed in order to extract the position of the first peak in the structure factor *S*(*q*) as follows: A SAXS form factor was fitted using the fuzzy sphere model (see SI for details) and based on the results from the SANS-ZAC analysis. The core and shell sizes were re-scaled based on the values obtained from SANS in order to adjust for the concentration-dependent particle size. All SAXS curves were thereafter fitted using the fuzzy sphere model, excluding the peak regime, to extract a concentration dependent normalisation factor for the intensity. The high *q*-range of the concentration dependent SAXS data were separately fitted using a Lorentzian function. The re-scaled core and shell sizes together with the intensity normalisation factor and the Lorentzian fit were used to regenerate concentration dependent form factors for extraction of the *S*(*q*) from the SAXS data using *S*(*q*) ~ *I*(*q*)/*CP*(*q*). This procedure corrects for the influence of the form factor on the overall intensity, i.e. for the small but systematic difference between the locations of the peak in *I*(*q*) and *S*(*q*), respectively. Note, however, that this effect is small, and using the location of the peak in *I*(*q*) directly would result in an overestimation of the center-center distance between particles *a*_*s*_ by a few percent only.

**Table 3:** Summary of the properties of the Hm-Dm mixture of microgels obtained from SANS experiments under ZAC conditions at all concentrations investigated.

*C* [%*wt*]	*ϕ*_*eff*_	*R*_*SANS*_ [*nm*]	2*σ*_*shell*_ [*nm*]	2*σ*_*shell*_/*R*_*SANS*_	*PD*
2	0.24	86.5 ± 0.3	31.6 ± 0.2	0.37	0.15 ± 0.02
4	0.48	84.7 ± 0.3	30.2 ± 0.2	0.36	0.12 ± 0.02
6	0.72	82.8 ± 0.3	28.4 ± 0.2	0.34	0.12 ± 0.02
8	0.96	82.1 ± 0.3	27.6 ± 0.2	0.34	0.14 ± 0.02
10	1.2	79.0 ± 0.3	26.0 ± 0.2	0.33	0.15 ± 0.02

Shown are the number-averaged values of the overall radius *R*_*SANS*_ = *R* + 2*σ*_*shell*_, the fuzzy shell 2*σ*_*shell*_, the size ratio 2*σ*_*shell*_/*R*_*SANS*_, and the polydispersity *PD* as obtained from a fit of the fuzzy sphere model to the experimental data for *T* = 27 °C.

### Viscometry

Capillary viscometry measurements were performed using a micro-Ostwald viscometer with a capillary diameter of 0.43 mm and a total volume of 2 ml. We determine the effective volume fraction *ϕ*_*eff*_ from the concentration dependence of the relative viscosity *η*_*r*_ = *η*_0_/*η*_*s*_, where *η*_0_ is the zero shear viscosity of the sample and *η*_*s*_ the viscosity of the pure solvent^3, 11^, together with the Batchelor equation for effective hard spheres^29^

4 $${\eta }_{r}=1+2.5{\varphi }_{eff}+5.9{\varphi }_{eff}^{2}=1+2.5kC+5.9{(kC)}^{2}$$

where *ϕ*_*eff*_ = *kC*, *C* is the microgel weight fraction and *k* the shift factor. The relative viscosity was obtained as a function of concentration at different temperatures, and the results for the different particles are shown in Fig. S1 in SI. The results from fitting the data to equation (4) are summarised in Table 4.

## Electronic supplementary material

Supplementary Information
