# Supplementary material for: Interpenetration of polymeric microgels at ultrahigh densities
Source: Sci Rep. 2017 May 3;7:1487. doi: 10.1038/s41598-017-01471-3 (PMC5431201; doi:10.1038/s41598-017-01471-3)
Supplement: Supplementary file 1 — Supplementary Information [file 41598_2017_1471_MOESM1_ESM.pdf]

Interpenetration of polymeric microgels at ultrahigh densities - Supplementary Information

Priti .S. Mohanty[1,2], Sofi Nöjd[1], Kitty van Gruijthuijsen[3], Jérôme J. Crassous[1], Marc Obiols-Rabasa[1], Ralf Schweins[4], Anna Stradner[1], and Peter Schurtenberger[1]

[1] *Physical Chemistry, Department of Chemistry, Lund University, 22100 Lund, Sweden*

[2] *School of Applied Sciences, KIIT University, Bhubaneswar 751024, India*

[3] *Adolphe Merkle Institute, University of Fribourg, 1700 Fribourg, Switzerland and*

[4] *Large Scale Structures Group, Institut Laue-Langevin, F-38042 Grenoble CEDEX 9, France*

(Dated: April 23, 2017)

Viscometry

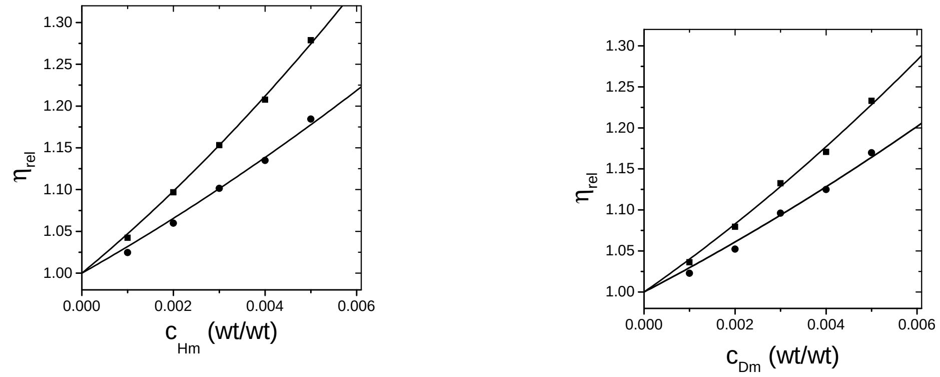

FIG. S1: Capillary viscometry data. The relative viscosity was extracted by normalizing the kinematic viscosity with that of the solvent for different concentrations, and thereafter fitting the data to the Batchelor equation (see main text) at different temperatures. Each measurement is the average of 5 measurements with a relative standard deviation of less than 1%. Left: Data obtained for Hm. This results in shift factor of $k = 18.1 \pm 0.2$ (filled squares) at 15.1°C and $k = 12.4 \pm 0.3$ (filled circles) at 27.1°C. Right: Data obtained for Dm. This results in shift factor of $k = 15.5 \pm 0.2$ (filled squares) at 15.1°C and of $k = 11.6 \pm 0.3$ (filled circles) at 27.1°C.

SANS measurement of dilute microgels

In the analysis of the SANS data we profit from the work of Stieger et al. [1], where the authors developed a model that takes into account the inhomogeneous nature of the microgels. A particle then consists of a dense core with high cross-linking density and a fuzzy shell with decreasing cross-link density. The radius of the high cross-link density core is given by R, and the overall particle size is given by $R_{SANS} = R + 2\sigma_{shell}$, where $2\sigma_{shell}$ describes the width of the fuzzy shell. R_{SANS} can be directly related to the average hydrodynamic radius R_h obtained by DLS [1].

We use an analogous approach where we also incorporate polydispersity using a Gaussian size distribution $D(R, \langle R \rangle, \sigma_{poly})$ characterized by an average radius $\langle R \rangle$ and a polydispersity σ_{poly}. The data is then analyzed using eqn. 1,

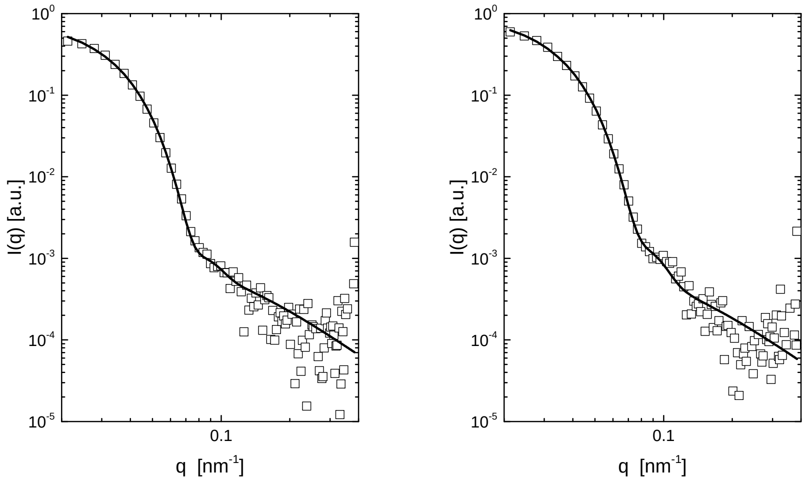

FIG. S2: Scattering intensity $I(q)$ as function of q at $T = 16.4°$C for the hydrogenated (Hm) (left) and deuterated (Dm) (right) particles at a low effective volume fraction ϕ_{eff} of 0.09 for Hm and 0.08 for Dm. Also shown are the corresponding fits to eqn. 1

$$\frac{d\sigma}{d\Omega}(q) = n_p \Delta\rho^2 \int_0^\infty \int_0^\infty R_{res}(\langle q\rangle, q) D(R, \langle R\rangle, \sigma_{poly}) V(R)^2 P_{fuzzy}(q,R)\, dR\, dq + I_{fluct}(q) + c_{back} \tag{1}$$

where $V(R)$ is the volume of polymer in a particle with core radius R, $P_{fuzzy}(q,R)$ is the form factor of a microgel particle with radius R taking into account the core-fuzzy shell structure, $R_{res}(\langle q\rangle, q)$ describes the resolution function of the SANS instrument, $I_{fluct}(q)$ describes the scattering contributions arising from fluctuations of the microgel network and is represented by a Lorentzian function of the form $I_{fluct}(q) = I_{fluct}(0)/(1+\xi^2 q^2)$, and c_{back} is a constant background used to account for residual incoherent background. While we expect that ξ reflects the average cross-link density of the particles, its contribution becomes significant only at large q-values. In this q-range the incoherent background in the SANS experiments is large, leading to a significant statistical error for the data and the resulting ξ parameter, which could thus not be extracted reliably from the analysis of the small-angle scattering data. Therefore, we have kept $\xi = 10$ nm constant in our final data analysis, consistent with the results from earlier investigations with particles of comparable cross-linking density.

The form factor the form factor $P_{fuzzy}(q,R)$ is given by

$$P_{fuzzy}(q,R) = \left[\frac{3(sin(qR) - qRcos(qR))}{(qR)^3} exp\left(-\frac{(\sigma_{shell}q)^2}{2} \right) \right]^2 \tag{2}$$

where R is the particle core size, $R = R_{box} + 2\sigma_{shell}$, R_{box} the initial box profile with constant scattering length density of the core and $2\sigma_{shell}$ the shell thickness. The particle size, R_{tot}, is defined as $R_{tot} = R + 2\sigma_{shell}$.

The particle size and shape in the dilute limit was determined using SANS in the swollen limit at a temperature of $T = 15°C$ as an additional consistency test. The resulting SANS data and fitted curves are shown in Fig. S2, and we obtain values of the number averaged $R_{SANS} = 95.7$ nm and 93.4 nm, and a shell with $2\sigma = 34$ nm and 36.3 nm for the Hm and Dm particles, respectively.

Contrast Variation

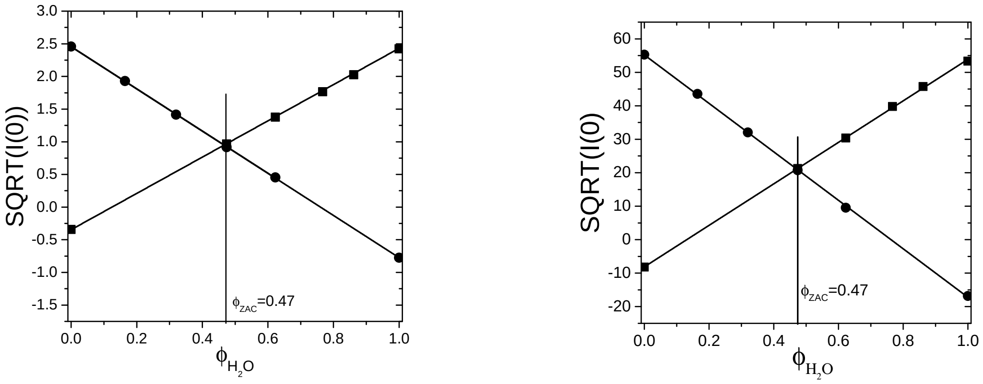

FIG. S3: Contrast variation experiments performed with hydrogenated (Hm, filled circles) and deuterated (Dm, filled squares) microgels as a function of the solvent ratio given by the volume fraction of H_2O, ϕ_{H_2O}. Left: Data obtained at 16.4°C. Right: Data obtained at 27.1°C.

SANS-ZAC and SAXS experiments at $T = 27°C$

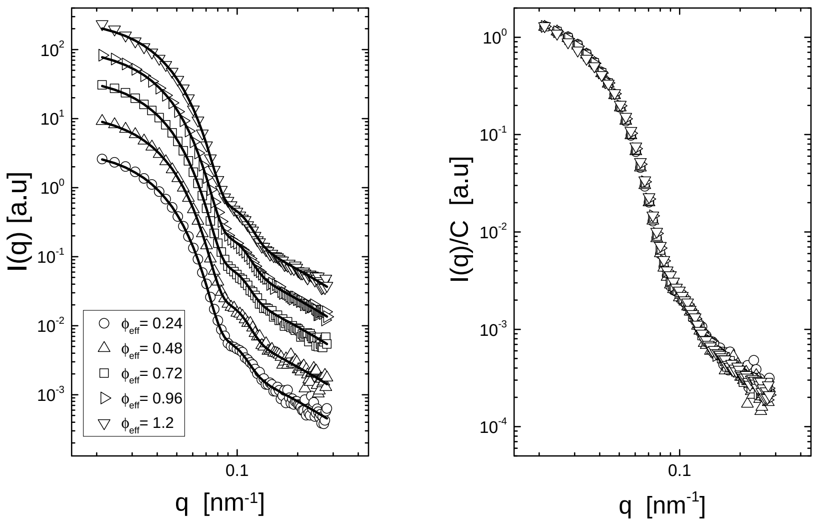

FIG. S4: Examples of the scattered intensity $I(q)$ as a function of the scattering vector q for a 50-50 mixture of Hm and Dm particles at $T = 27.1°C$ in a ZAC solvent mixture for different values of the effective volume fraction $0.24 \leq \phi_{eff} \leq 1.2$ (left). The solid lines are the fits of the form factor for fuzzy spheres to the experimental data. Also shown is the normalised scattering intensity $I(q)/C \sim \frac{d\sigma}{d\Omega}(q)$ as a function of the scattering vector q for the same samples (right).

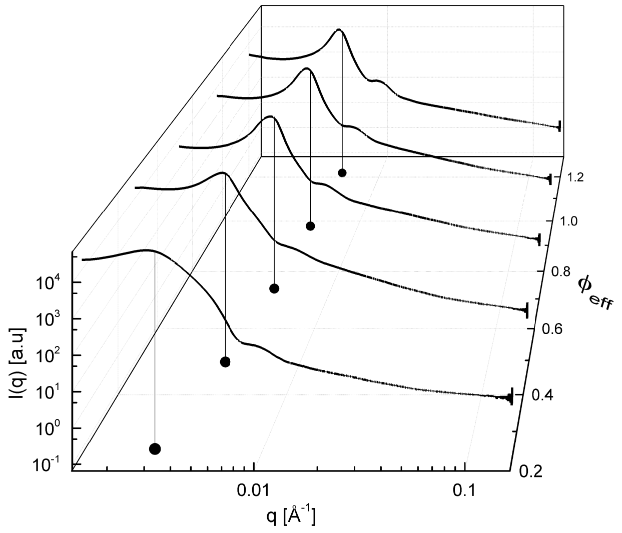

FIG. S5: Examples of the scattered intensity $I(q)$ as a function of the scattering vector q from SAXS experiments with a 50-50 mixture of Hm and Dm particles at $T = 27°C$ in a ZAC solvent mixture for different values of the effective volume fraction. The position of the maximum of the resulting structure factor peak is also indicated.

SANS tracer experiments

We have also investigated the behaviour of the Hm particles separately by performing tracer measurements, where a small number of Hm particles was added to a majority phase of Dm particles under solvent conditions where the Dm particles were completely matched, i.e. where the SANS experiment again provided the form factor of the Hm particles only. Experiments were carried out at the instrument SANS-1 at the Swiss neutron source SINQ, Paul Scherrer Institut (PSI), Switzerland. Sample-to-detector and collimation distances of 18 m and a wavelength of 0.8 nm was used. The scattering length density of the Dm particles was determined by carrying out SANS measurements in six deuterated microgel samples, each with a concentration of 0.32 wt% and with a varying volume % of H_2O (100, 85, 75, 60, 45 and 0). The match point was found to be at 13 vol% of H_2O. For the tracer experiments, six samples were prepared by adding a fixed amount of tracer particles (Hm) to different concentration of Dm-microgels (see Table S1) at a solvent ratio of 13 vol% of H_2O. The volume fraction of tracers in each sample is 0.06. The shift factor k obtained from capillary viscometry studies of Dm particles is $k = 20$. The Hm and Dm particles were furthermore characterised by DLS, and a summary of their swelling behavior

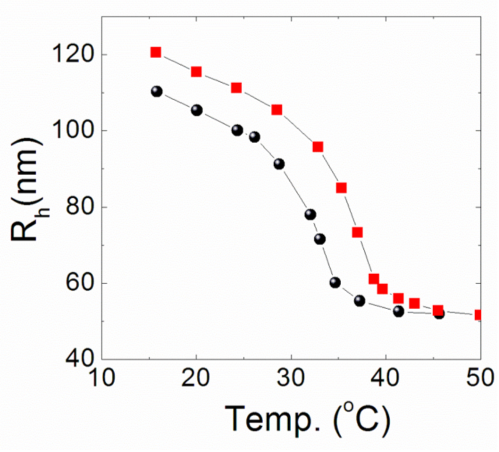

FIG. S6: Temperature dependence of the hydrodynamic radius R_h for the deuterated and hydrogenated particles in H_2O as a function of temperature for deuterated (red squares) and hydrogenated (black circles) particles.

is given in Fig. S6

The tracer measurements are summarized in Fig. S7. Shown are the individual form factors measured and the fit using the fuzzy sphere model (left), and the resulting normalised particle sizes R_{SANS}/R_0 as a function of effective volume fraction ϕ_{eff}.

TABLE S1: Summary of the properties of the Hm tracer particles embedded in a dense suspension of Dm particles with different concentrations obtained from SANS experiments where the Dm particles are matched against the solvent (see text for details). Shown are the number-averaged values of the overall radius $R_{SANS} = R + 2\sigma_{shell}$, the fuzzy shell $2\sigma_{shell}$, the size ratio $2\sigma_{shell}/R_{SANS}$, and the polydispersity PD as obtained from a fit of the fuzzy sphere model to the experimental data for $T = 15$ °C.

C $[g/g]$	ϕ_{eff}	R_{SANS} $[nm]$	$2\sigma_{shell}$ $[nm]$	$2\sigma_{shell}/R_{SANS}$	PD
0.028	0.56	102.1 ± 0.1	32.7 ± 0.3	0.32	0.08
0.0559	1.12	100.9 ± 0.1	32.3 ± 0.3	0.32	0.08
0.0651	1.3	100.4 ± 0.1	31.8 ± 0.3	0.32	0.08
0.0738	1.48	100.1 ± 0.1	31.7 ± 0.3	0.32	0.08
0.0921	1.84	98.6 ± 0.1	30.4 ± 0.3	0.31	0.08
0.1063	2.13	96.1 ± 0.1	28.0 ± 0.3	0.29	0.08

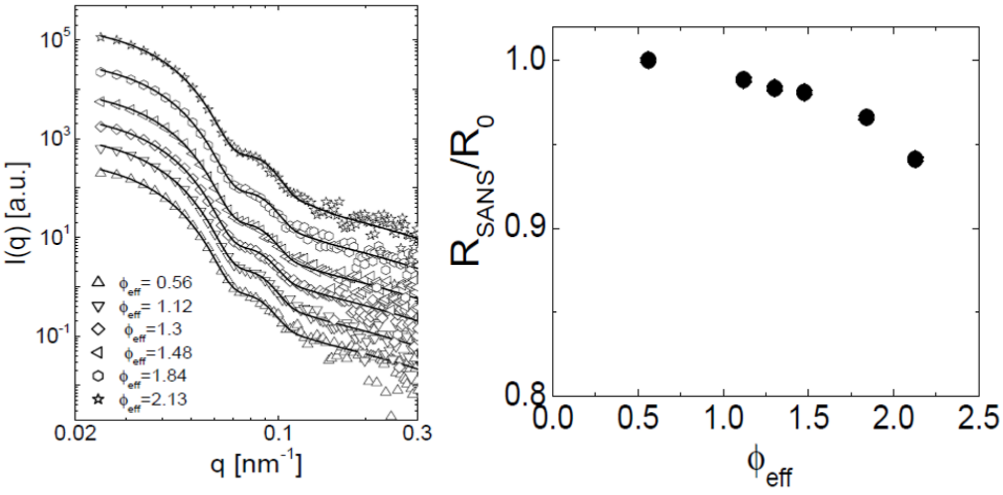

FIG. S7: Left: Examples of the scattered intensity $I(q)$ as a function of the scattering vector q for the tracer experiment, where a small amount of Hm is measured in a dense suspension of Dm particles at $T = 15°$C in a solvent mixture that matches the Dm particles for different values of the effective volume fraction $0.56 \leq \phi_{eff} \leq 2.13$. The solid lines are the fits of the form factor for fuzzy spheres to the experimental data. Right: Normalised overall dimensions $R_{SANS}/R_{SANS,0}$ as a function of ϕ_{eff} obtained from SANS tracer measurements at $T = 15°$C.

[1] M. Stieger, W. Richtering, J. S. Pedersen and P. Lindner, *J. Chem. Phys.*, 2004, **120**, 6197.
